# License: A, Diploma; B, Examination

**Published:** 1901-07

**Authors:** J. A. Libbey

**Affiliations:** Pittsburg, Pa.


### LICENSE: A, DIPLOMA; B, EXAMINATION.[1]

BY J. A. LIBBEY, D.D.S., PITTSBURG, PA.

A COLLEGE chartered by the State for the purpose of educating students, whether it be a classical course or upon some special branch, employing well-qualified teachers, who are thoroughly in earnest in their work and loyal to their profession, and are desirous to see only those who are thoroughly competent enter it, who count the qualities of the student of more value than the profits of the institution, should have no other requirement for entering upon professional work than a diploma from such a school.

It is evident that the schools are not all conducted on a basis like this, for the reason that there must be sufficient funds to defray

[1] Abstract of paper read before the American Medical Association, Section on Stomatology, St. Paul, Minn., June 4, 5, 6, and 7, 1901.

all expenses, including the assistants' salaries, and sufficient dividend to justify the faculty or incorporators for the valuable time taken from their practice; not considering the added tone and dignity of being called "professor" (which is frequently used for profit).

The question arises, "How many students can we get at $—— apiece?" If one hundred are secured, it will be difficult to get sufficient clinics for demonstration; how will this be accomplished? The usual way is to urge the students to solicit, not among the poor and needy, but among their own class of people, who are usually able to pay a fair fee.

"A chain is no stronger than the weakest link." If license by diploma is accepted, then the poorest school sets the standard for all. Hence the necessity of licensing by examination.

The necessity for examination is not a new idea; it can scarcely be called a modern one, for in 1700 a French writer urges the necessity of examinations in France, for the practice of dentistry.

The history of reform in the dental profession in England, by Hill, conclusively shows not only the necessity of requiring examinations, but also the advancement from a scientific and professional stand-point, by which the people were the beneficiaries. On page 86 he quotes the remarks of the president of the Odontological Society (the late S. Cartwright, Esq.). "It cannot be doubted that a liberal education is of the greatest advantage to those engaged in practice, and the more education is extended in all ranks of society the more it becomes necessary that the members of our profession qualify themselves as highly as they can, for those who employ the services in these days have a right to look, and do look, to the qualities of the mind as well as mechanical adroitness of the fingers."

The main object of the above society was to unite members of the dental profession into a recognized body, and provide a means of professional education and examination.

In nearly every criticism on dental legislation, or State board of examiners, the author gives the impression that the laws were passed for the benefit of the profession; if such were the case, they should be repealed, as class legislation is in direct opposition to the principles of our government. The laws were made for the people.

The medical laws are similar to ours, and the profession of law requires the same. In the writer's State (Pennsylvania) a law student must pass a preliminary, and after graduation must pass an examination before a board appointed by the court, the only persons competent to appoint that board.

The best means possible should be adopted to test the qualifications of the aspirant to professional practice, and the most important is a judicious selection of competent examiners. As stated above, the court appoints the examiners for the law; they are the most competent to perform that duty, but would prove themselves unqualified were it required of them to appoint a medical board.

No one is competent to appoint a board for professional examinations except members in good standing in that profession. The difficulty of securing competent men for examiners in our profession arises from the necessity of their being appointed by the governor, thus making them officers of the State, and he, being authorized to appoint, may be ill advised in the selection.

As the court is the representative body of the profession of law, and is competent, the only representative body of the dental or the medical profession in the State is the State society. It should be a State society in fact as well as in name, which is not the case in some States. It must be chartered by the State, and to be a representative body must be made up of representatives (or delegates) from the local societies throughout the State. Act of 1897 (Pennsylvania), gives the power of appointment to the governor, and the selection to the State society (which is a representative body): "Section 5. The Pennsylvania State Dental Society may, at its annual meeting in one thousand eight hundred and ninety-seven, and annually hereafter at said meeting, select as nominees the names of double the number of examiners required, who shall be members in good standing of the Society, and transmit such names to the governor under its seal and signed by its secretary. From this list of nominees the governor shall, during the month of August, Anno Domini one thousand eight hundred and ninety-seven, appoint a board of dental examiners. In case of failure of the said Pennsylvania State Dental Society to submit such list, as aforesaid, the governor shall appoint members in good standing of the said Society without other restrictions. Each one of the said appointees must be a registered, *bona fide* practitioner of dentistry in good standing, and shall have practised dentistry under the laws of this State for a period of not less than ten years. No member of a dental college faculty shall be eligible to appointment upon the State Board of Dental Examiners, but this shall not apply to membership in the Dental Council. The governor shall fill vacancies by death or otherwise for the unexpired term of said examiners, from the list of names submitted to him by the dental society."

Since the present law went into effect, eight hundred and twenty-three applicants have been examined (these were graduates from eighteen different schools); of this number, seven hundred and eleven passed successfully and received licenses from the Dental Council; one hundred and twelve failed to receive the required average, a fraction over thirteen and one-half per cent.

"The measure had an *ex post facto* character, inasmuch as when it was passed many students had already spent two years in the dental schools, and in recognition of this fact the board of examiners has been lenient in the examination of applicants. This has no doubt aided in fostering good will towards the law. The influence of the work of the board of examiners has been beneficial, in that it has given a stimulus to the teaching in the schools, and has induced stricter scrutiny of the acquirements of candidates for diplomas. Formerly students often sought the schools from which they could be graduated most easily, but now they prefer those that require a higher standard." [1]

If the board had marked the first two years, as they have since, the percentage of failures would have been doubled, as fully that number barely passed the required average (seventy-five per cent.). When the profession and the people realize the condition that now exists, there will be no difficulty in getting laws passed that would be a real protection against quackery, and contribute to hasten the time when the stomatologist will take the degree of M.D., leaving the name "dentist" to designate those who do only mechanical work.

---

[1] Extract from report to the governor.

